# Dépistage de l'atteinte oculaire chez les enfants sourds

**DOI:** 10.11604/pamj.2019.33.174.17771

**Published:** 2019-07-05

**Authors:** Belghmaidi Sarah, Belhoucha Btissam, Hajji Ibtissam, Rochdi Youssef, Albab Nabil, Nouri Hassan, Aderdour Lahcen, Raji Abdelaziz, Moutaouakil Abdeljalil

**Affiliations:** 1 Service d'Ophtalmologie, CHU Mohammed VI, Marrakech, Maroc; 2 Service d'ORL, CHU Mohammed VI, Marrakech, Maroc

**Keywords:** Syndromes de surdité, syndromes de cécité, atteinte oculaire, enfants, Deafness syndrome, blindness syndrome, ocular involvement, children

## Abstract

L'association de la surdité aux troubles visuels est fréquente. Ces troubles vont de simple anomalie de la réfraction jusqu'à la maladie grave qui peut constituer un handicap. D'où l'intérêt d'un dépistage précoce. L'objectif de cette étude est de montrer l'importance de la collaboration multidisciplinaire et la nécessité de l'examen ophtalmologique chez chaque enfant présentant une surdité à travers cette étude prospective. Il s'agit d'une étude prospective monocentrique colligeant 200 enfants suivis pour hypoacousie de janvier 2014 à janvier 2015. Chaque enfant a bénéficié d'un examen ophtalmologique complet; examen ORL; et un examen général. Cent cinquante-cinq dossiers ont été colligés. Une atteinte oculaire était constatée chez 47 patients soit 30,4% des enfants. Elle est bilatérale chez 45patients. Les principales étiologies étaient syndromiques (syndrome d'Usher (8cas), syndrome de Waardenbourg (5 cas), syndrome d'Alport (3 cas), syndrome de Wolfram (2 cas), syndrome de Goldenar (3 cas), syndrome de Cogan (3 cas), syndrome de Fracheschetti (1 cas), syndrome de Charge (1 cas), syndrome otomandibulaire (1 cas), syndrome de Stickler (1 cas), syndrome d'Alstrom (1 cas), syndrome de Refsum (1 cas), syndrome de Susac (1 cas) et KID syndrome (1 cas)). Le dépistage de l'atteinte oculaire a permis de raccourcir le délai d'attente pour implant cochléaire de 9 mois à 3 mois. Les atteintes oculo auditives sont très nombreuses du fait de la similitude embryologique et cellulaire de ces deux organes, notamment la rétine et l'oreille interne. Le diagnostic de ces atteintes est facilité par l'existence d'une dysmorphie faciale, en revanche, il reste difficile lorsqu'il existe que les atteintes neurosensorielles visuelles et auditives. La précocité du diagnostic des atteintes oculo auditives permet un meilleur développement psychomoteur et une insertion sociale optimale. Donc la prise en charge pluridisciplinaire précoce est nécessaire afin de permettre la meilleure rééducation psychomotrice, orthophonique et visuelle.

## Abstract (en)

The association between deafness and visual disorders is frequent. These disorders range from simple refractive disorder to severe disease that can lead to disability. Hence the interest of early screening. This study aims to highlight the importance of multidisciplinary management and of the need for ophthalmological examination in each deaf child. We conducted a prospective monocentric data collection from medical records of 200 children followed for hypoacousia from January 2014 to January 2015. Each child underwent complete ophthalmological examination, ENT examination and clinical examination. Data from 155 medical records were collected. Ocular involvement was found in 47 patients, reflecting a rate of 30.4%. Bilateral involvement was found in 45 patients. The main syndromic causes were: Usher syndrome (8 casess), Waardenbourg syndrome (5 cases), Alport syndrome (3 cases), Wolfram syndrome (2 cases), Goldenhar syndrome (3 cases), Cogan syndrome (3 cases), Franceschetti-Kleinsyndrome (1 case), Charge syndrome(1 case), otomandibular syndrome (1 case), Stickler syndrome(1 case), Alström syndrome (1 case), Refsum disease (1 case), Susac syndrome (1 case) and KID Syndrome (1 case). Screening for ocular involvement allowed to shorten the average length of cochlear implantation from 9 months to 3 months. There are numerous ocular and auditory involvements because of the embryological and cellular similarities of these two organs, including the retina and the inner ear. The diagnosis of these involvements is facilitated by the presence of facial dysmorphism; on the other hand, diagnosis is difficult when there are visual and auditory sensorineural involvements. Early diagnosis of ocular and auditory involvements allows for best psychomotor development and optimal social inclusion. Therefore multidisciplinary management is necessary to allow for the best psychomotor, orthophonic and visual rehabilitation.

## Introduction

La déficience auditive est le déficit sensoriel le plus fréquent à la naissance. C'est un problème de santé publique en raison des chiffres élevés de prévalence et de la répercussion sur le développement de la communication, sur la scolarité et l'insertion sociale ultérieure de l'enfant. Un enfant sur mille naît sourd profond, ce qui représente environ 25% des surdités présentes à la naissance. À 3 ans, la prévalence des surdités sévères et profondes est de 3/1000 [1-3]. Les troubles visuels et neurovisuels sont fréquemment associés à la surdité. Ces troubles vont du simple trouble de la réfraction jusqu'à la maladie grave qui peut constituer un handicap. Le dépistage des troubles visuels chez les enfants sourds ou malentendants fait appel à la compétence de l'ensemble des professionnels de santé au contact de l'enfant car seule la précocité du dépistage et du diagnostic est gage de la réussite thérapeutique et permet d'éviter un polyhandicap sensoriel [1-5]. L'objectif de travail est de montrer l'importance de la collaboration multidisciplinaire, et la nécessité de l'examen ophtalmologique chez chaque enfant présentant une surdité à travers cette étude prospective.

## Méthodes

Il s'agit d'une étude prospective monocentrique colligeant 200 enfants suivis pour hypoacousie de janvier 2014 à janvier 2015. Une consultation multidisciplinaire commune ophtalmologique, ORL et pédiatrique a été créée à raison d'une fois par semaine afin de mieux prendre en charge les enfants ayant plusieurs atteintes neurosensorielles. Chaque enfant a bénéficié d'un examen ophtalmologique complet; examen ORL; et un examen général. Les données ont été collectées sur une fiche standardisée incluant à la fois l'atteinte auditive qu'oculaire et précisant les critères suivants: L'âge, le sexe, le déroulement de la grossesse et de l'accouchement, l'examen ophtalmologique avec photographies du segment antérieur et postérieur, l'examen ORL avec audiogramme, les examens paracliniques: PEA, ERG, EOG, et la prise en charge oculaire et auditive. Tous les malades ayant un retard mental ont été exclus de l'étude: les infirmités motrices cérébrales (30 cas) et les trisomiques (15). Nous avons retenu dans cette étude 155 enfants. Pour l'étude génétique, nous avons dressé les arbres généalogiques des patients ayant une atteinte syndromique.

## Résultats

**Figure 1:**
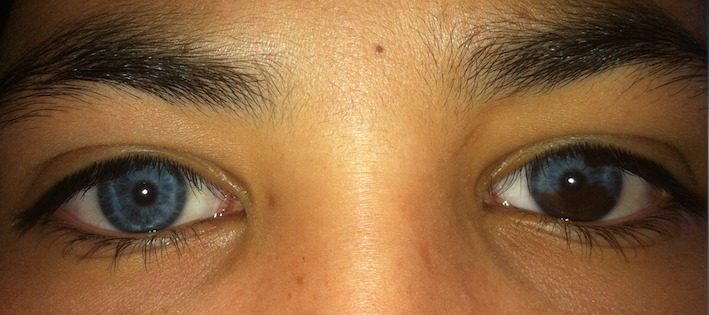
Hétérochromie irienne chez une fillette qui présente un syndrome de Waardenbourg

**Figure 2:**
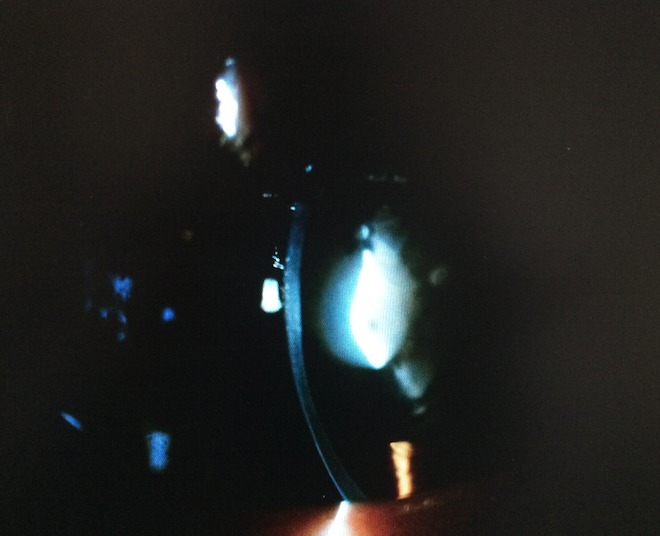
Lenticone avec cataracte dans le cadre du syndrome d'Alport

**Figure 3:**
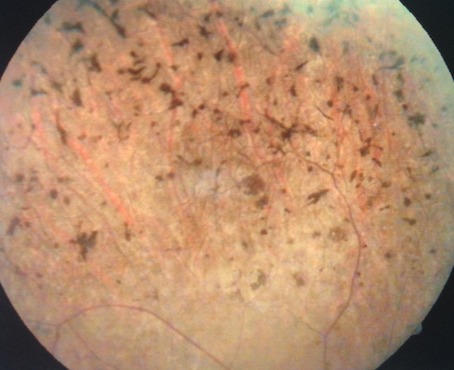
Fond de l'œil objectivant une rétinopathie pigmentaire dans le cadre d'un syndrome d'Usher

**Figure 4:**
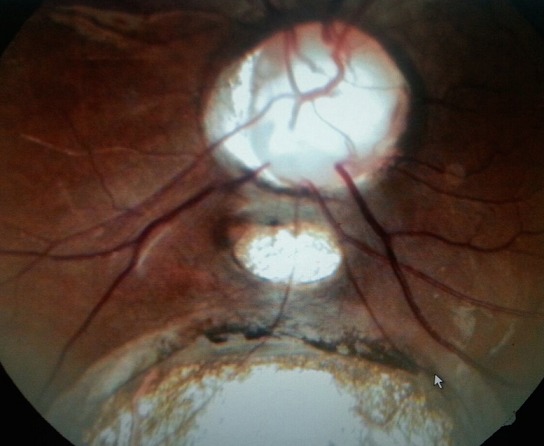
Colobome infra papillaire

Cent cinquante-cinq dossiers ont été colligés, l'âge moyen de nos patients était 7 ans (allant d'un an à 15 ans), la répartition selon le sexe était 96 filles et 59 garçons. L'examen oto-rhino-laryngologique a objectivé à l'otoscopie un tympan d'aspect normal chez tous les patients, une audiométrie comportementale ou tonale en fonction de l'âge a été réalisée. Des PEA ont été demandés chez 135 patients qui ont objectivé une surdité de perception chez 127. La perte moyenne était de 73db [40 - 120]. Une atteinte oculaire était constatée chez 47 patients soit 30,4% des enfants. Elle est bilatérale chez 45 patients. Les principales étiologies étaient syndromiques (syndrome d'Usher (8cas), syndrome de Waardenbourg (5 cas), syndrome d'Alport (3 cas), syndrome de Wolfram (2 cas), syndrome de Goldenar (3 cas), syndrome de Cogan (3 cas), syndrome de Fracheschetti (1 cas), syndrome de Charge (1 cas), syndrome otomandibulaire (1 cas), syndrome de Stickler (1 cas), syndrome d'Alstrom (1 cas), syndrome de Refsum (1 cas), syndrome de Susac (1 cas) et KID syndrome (1 cas)). Suivies des étiologies métaboliques (7 cas de muccopolysaccahridose), puis infectieuse: syphilis (2 cas), Vogt koyanakiharrada (1 cas), rubeole (3 cas) et l'histiocytoselangehansienne (1 cas). La meilleure acuité visuelle corrigée moyenne était de 5/10 allant de 1/10 à 10/10. Une atteinte cornéenne a été objectivée chez 14 patients: 7 avaient des opacités nuageuses stromales antérieures, 5 kératites interstitielles, et deux dermoides de limbe. L'iris a été touché chez 5 cas: hétérochromie chez 4 (Figure 1) et colobome chez un. Quatre enfants avaient une atteinte cristallinienne, un lenticône antérieure était trouvé chez deux enfants (Figure 2), et une cataracte chez trois. Une atteinte rétinienne était retrouvée chez 16 patients: rétinopathie pigmentaire chez 9 (Figure 3), une atrophie optique chez deux, une rétine albinoide chez 4 et une maculopathie chez un cas et un colobome infra papillaire chez un patient (Figure 4). L'examen des annexes a objectivé un ectropion chez un enfant avec une dystopiecanthale externe, une dacryocystite chez un, et un dystopie orbitaire chez un cas. Un strabisme a été trouvé chez 5 enfants. Les patients ayant une atteinte oculaire avaient une surdité de perception dans 42 cas dont 33 cas de surdité sévère à profonde et 5 cas une surdité de transmission. Les caractéristiques cliniques de chaque pathologie sont résumées dans le Tableau 1. Sur le plan thérapeutique, une chirurgie de cataracte a été réalisée chez trois enfants avec implantation dans un délai moyen de deux semaines, une greffe de cornée chez deux, Une exérèse du dermoide a été faite chez deux et une cure d'ectropion chez un cas. Un traitement optique a été proposé chez 30 enfants par des verres correcteurs chez 28 et lentilles rigides perméables au gaz chez deux. Tous ces enfants ont bénéficié d'une réeducation orthoptique avec prise en charge de l'amblyopie. La prise en charge ORL a consisté en 5 cas d'appareillage auditif, et 20 cas d'implant cochléaires. Un bilan d'implantation pour les autres patients est en cours (Tableau 2).

**Tableau 1:** Les caractéristiques cliniques des maladies et syndromes oculo auditifs

Pathologie	Atteinte oculaire	Atteinte ORL	Atteinte générale
Syndrome usher (8 cas)	Rétinopathie pigmentaire (figure 1)	Surdité de perception	
Syndrome d’alport (3 cas)	Lenticone antérieur (figure 2) Cataracte Maculopathie d’alport (1 cas)	Surdité de perception	Insuffisance rénale terminale (1 cas)
Syndrome de Waardenbourg (5 cas)	Achromie irienne (1 cas) Hétérochromie irienne (3 cas) : (figure 3) Fond d’œil albinoide	Surdité de perception	Elargissement de la base du nez (1 cas) Hypertrichose soucilière (1 cas).
Syndrome de Wolfram (2 cas)	Atrophie optique (2 cas)	Surdité de perception	Diabète insulinodépendant
Syndrome de Goldenar (3 cas)	Dermoide du limbe (2 cas)	Surdité de transmission Enchondrome prétragiens microtie	Atteinte vertebrale (1 cas)
Syndrome de Franceschetti (1cas)	Dysplasie orbitaire Dystopie canthale externe Dacryocystite chronique Ectropion	Surdité de transmission	
Syndrome de CHARGE (1 cas)	Colobome irien Colobome infrapapillaire (figure 4)	Surdité de transmission	Malformation cardiaque Retard mental
Syndrome otomandibulaire (1 cas)	Dystopie orbitaire (1 cas)	Surdité de transmission Hypoplasie mandibulaire	
KID syndrome (2 cas)	Kératite néovascularisation	Surdité de perception	Erythrokératodermie
Syndrome de stickler (1 cas)	Cataracte Myopie axile	Surdité de perception rétrognatisme	hyperlaxité
Syndrome d’alstrom (1 cas)	Rétinopathie pigmentaire	Surdité de perception	Obésité, diabète
Syndrome de Refsum (1 cas)	Rétinopathie pigmentaire	Surdité de perception	Ataxie cerebelleuse, ichtyose
Syndrome cogan (3 cas)	Kératite interstitielle	Surdité de perception	Cardiomyopathie (1 cas) Arthralgies (1 cas)
Syndrome de susac (1 cas)	Occlusions de multiples branches de l’artère centrale de la rétine	Surdité de perception	céphalées
Mucopolysaccharidose (7 cas)	Dépots floconneux stromaux antérieures	Surdité de perception	Retard staturo pondéral Dysmorphie faciale
Syphilis (2 cas)	Kératite interstitielle	Surdité de perception	
VKH (1 cas)	Uveite postérieure Poliose	Surdité de perception	Vitiligo alopécie
Rubéole (3 cas)	Cataracte congénitale (2 cas) Kératite (1 cas)	Surdité de	
Histiocytose langerhansienne (1 cas)	Dystopie orbitaire Lyse du plancher orbitaire	Surdité de perception Lyse de la pyramide pétreuse	Hépatomégalie Pancytopénie splénomégalie

**Tableau 2:** Le type et le délai de la prise en charge ORL

	Surdité de perception	Surdité de transmission	Délai de la prise en charge	Implant cochléaire		Appareillage auditif
				implantés	en cours	
Les patients n’ayant pas d’atteinte oculaire	85	23	09 mois	09	30	23
les patients ayant une atteinte oculaire	42	5	03 mois	20	21	05

## Discussion

Notre étude a démontré l'importance d'un dépistage systématique de l'atteinte oculaire chez les enfants présentant une surdité afin d'améliorer les conditions et le délai de prise en charge chez ces patients ayant une atteinte multisensorielle. La vision et l'audition sont deux sens essentiels complémentaires dans le développement psychomoteur d'un enfant; l'atteinte de l'un entraine le surdéveloppement de l'autre pour permettre une autonomie sociale. Les atteintes oculo auditives sont très nombreuses du fait de la similitude embryologique et cellulaire de ces deux organes, notamment la rétine et l'oreille interne [1-3]. Plus de 300 maladies et syndromes génétiques comportant une surdité sont actuellement décrits [5-8]. Selon notre étude 30,4% des enfants sourds présentent une anomalie ophtalmologique. Le diagnostic des syndromes oculo-auditifs est facilité par l'existence d'une dysmorphie faciale, en revanche, il reste difficile lorsqu'il existe que les atteintes neurosensorielles visuelles et auditives [9-12]. Notre étude a permis le dépistage de l'atteinte oculaire chez 31% des enfants grâce à la création d'une consultation multidisciplinaire. 48% de ces patients n'avaient pas de signes d'appels ophtalmologiques. Une prise en charge ophtalmologique et auditive précoce est essentielle pour insérer l'enfant dans la société. Les anomalies ophtalmologiques curables telles que la chirurgie de cataracte ont été traitées dans un délai moyen de 2 semaines. Une rééducation orthoptique a été prescrite chez tous ces enfants afin de rééduquer la fonction visuelle défaillante. Les enfants ayant une atteinte ophtalmologique d'aggravation progressive avec risque de cécité ont bénéficié d'une implantation cochléaire dans les brefs délais pour permettre de réhabiliter le plus rapidement possible la voie auditive, comme le syndrome d'Usher qui associe une surdité profonde, une rétinopathie pigmentaire progressive avec risque de cécité et une aréflexie vestibulaire. Dans ce cas l'indication d'implantation cochléaire est impérative du fait de le handicap auditif et visuel associés ne rendant possible qu'un projet oraliste de communication [13-15]. Dans notre série on a implanté 05 cas de syndrome d'Usher, les 2 autres sont en cours. 20 enfants parmi 42 ayant une surdité de perception ont bénéficié d'une implantation cochléaire. Les autres enfants sont en attente à cause du problème socio-économique source majeur du retard de prise en charge dans notre contexte. La rééducation orthophonique prolongée est essentielle pour développer l'utilisation corticale des informations auditives fournies par l'implant et entraîner la boucle audio-phonatoire. Il est recommandé de maintenir et développer toute forme de communication à partir des différentes afférences sensorielles, visuelles, tactiles, proprioceptives et auditives (communication multimodale) au cours des rencontres effectuées avec l'enfant et sa famille dans le cadre de l'intervention précoce [14, 16, 17]. Le dépistage précoce de ces troubles est essentiel par examen ophtalmologique détaillé, complet et qu'il faudra savoir répéter. Ce protocole de dépistage a permis de raccourcir le délai d'implantation de 9 mois à 3 mois chez quelques enfants [15-17].

## Conclusion

Le diagnostic des pathologies oculo auditifs est difficile, il nécessite une collaboration multidisciplinaire. La précocité du diagnostic permet un meilleur développement psychomoteur et une insertion sociale optimale. Donc la prise en charge pluridisciplinaire précoce est nécessaire afin de permettre la meilleure rééducation psychomotrice, orthophonique et visuelle, et éviter ainsi l'enfermement de l'enfant dans un mutisme total.

### Etat des connaissances actuelles sur le sujet

- La nature de l'atteinte oculaire dans les différents syndromes oculo auditifs;
- Les modalités diagnostiques et thérapeutiques des syndromes oculo auditifs.

### Contribution de notre étude à la connaissance

- C'est la première étude faite au CHU de Marrakech rapportant les difficultés diagnostiques et thérapeutiques de ces enfants sourds dans notre contexte.

## Conflits des intérêts

Les auteurs ne déclarent aucun conflit d’intérêts.
